# Phytochemical analysis and biological activities of essential oils extracted from *Origanum grossii* and *Thymus pallidus*: in vitro and in silico analysis

**DOI:** 10.1038/s41598-023-47215-4

**Published:** 2023-11-16

**Authors:** Hind Zejli, Aziza Fitat, Youssra Lefrioui, Farhan Siddique, Mohammed Bourhia, Fatima Zahra Bousseraf, Ahmad Mohammad Salamatullah, Hiba-Allah Nafidi, Amare Bitew Mekonnen, Abdelkader Gourch, Mustapha Taleb, Abdelfattah Abdellaoui

**Affiliations:** 1 Laboratory of Engineering, Electrochemistry, Modeling and Environment, Faculty of Sciences Dhar El Mahraz, Sidi Mohammed Ben Abdellah University, B. P. 1796, Fes-Atlas, Morocco; 2 Laboratory of Biotechnology, Health, Agrofood and Environment, Faculty of Sciences Dhar El Mahraz, Sidi Mohammed Ben Abdellah University, B. P. 1796, Fes-Atlas, Morocco; 3 Department of Pharmaceutical Chemistry, Faculty of Pharmacy, Bahauddin Zakariya University, Multan, 60800 Pakistan; 4 Department of Chemistry and Biochemistry, Faculty of Medicine and Pharmacy, Ibn Zohr University, 70000 Laayoune, Morocco; 5 Department of Food Science & Nutrition, College of Food and Agricultural Sciences, King Saud University, 11, P.O. Box 2460, Riyadh, 11451 Saudi Arabia; 6 Department of Food Science, Faculty of Agricultural and Food Sciences, Laval University, 2325, Quebec City, QC G1V 0A6 Canada; 7 Department of Biology, Bahir Dar University, P. O. Box 79, Bahir Dar, Ethiopia

**Keywords:** Drug discovery, Immunology, Plant sciences

## Abstract

The study aimed at investigating the phytochemical composition, antioxidant and antibacterial activities of essential oils (EOs) of *Origanum grossii* and *Thymus pallidus*. The selection of these plants for the study was driven by a comprehensive survey conducted in the Ribat Elkheir region of Morocco, where these plants are widely utilized. The results reflect the valorization of these plants based on the findings of the regional survey. The GC–MS phytochemical analysis revealed that the main constituents of the essential oil were carvacrol and thymol for *O. grossii* and *T. pallidus* respectively. Quantitative assays demonstrated that *O. grossii* exhibited higher levels of polyphenols (0.136 mg AGE/mg EO) and flavonoids (0.207 mg QE/mg EO) compared to *T. pallidus*. The DPPH assay indicated that *O. grossii* EOs possessed approximately twice the antiradical activity of *T. pallidus*, with IC_50_ values of approximately 0.073 mg/mL and 0.131 mg/mL, respectively. The antibacterial activity tests showed that both essential oils exhibited significant inhibition zones ranging from 26 to 42 mm against all tested bacterial strains. The MIC values varied among the bacteria, generally falling within the range of 0.31 to 2.44 µg/mL, demonstrating the potency of the EOs to serve as antibacterial. Molecular docking revealed that *O. grossii* and *T. pallidus* essential oils interact with antibacterial and antioxidant proteins (1AJ6 and 6QME). Key compounds in *O. grossii* include p-cymene, eucalyptol, and carvacrol, while *T. pallidus* contains potent chemicals like p-cymene, ɤ-maaliene, valencene, α-terpinene, caryophyllene, himachalene, and thymol. Notably, the most potent chemicals in *Origanum grossii* are *p*-cymene, eucalyptol, and carvacrol, while the most potent chemicals in *Thymus pallidus* are *p*-cymene, α-terpinene, and thymol. These findings suggest that these plant EOs could be used to develop new natural products with antibacterial and antioxidant activity.

## Introduction

Historically, naturally occurring substances have been utilized as food additives and have also been explored for their therapeutic potential. The properties which are domiciled in different parts of the plant often draw wide attention, with the essential oils (EO) of aromatic plants being a cynosure in this context^1,2^. These EOs often possess a surfeit of pharmacological properties including anticancer, anti-inflammatory, antioxidant, insecticidal, and antimicrobial properties among many others. Concomitant with the presence of these properties is the presence of an array of phytochemicals in EOs which confers these potentials. However, the phytochemical composition of EOs is affected by different factors including ecological (geographical origin, soil composition, climate condition), genotype (species, clone, cultivar, ecotype), and technical (elements relating to agriculture, forms of collecting, and crude material storage)^3,4^. Consequently, EO from similar plant species can express different features and phytochemical composition which could be dependent on or influenced by the previously mentioned factors.

The *Lamiaceae* family, which consists of a wide variety of herbs, plays a significant role in the Mediterranean region because of its well-known medicinal and aromatic qualities. Two notable members of this family, specifically *Origanum grossii* (*O. grossii)* and *Thymus pallidus* (*T. pallidus)*, epitomize the importance of this family. The Lamiaceae family is distinguished by a common physiological and morphological structure, with a notable feature being the presence of essential oils (EOs). These oils have attracted considerable interest due to their potential therapeutic properties^5^. The significance of investigating the phytochemical constituents of these herbs is highlighted by this shared characteristic.

The genus *Origanum* is widely recognized and utilized globally due to its numerous species that hold culinary and medicinal value^6^. However*, O. grossii* is considered a distinct entity that has received less attention due to its limited distribution within the Northwest (NW) region of Morocco^6,7^. The unique nature of its habitat is further emphasized by its preference for high elevations and humid climates within the Western Rif Mountain range^8^. The species’ specific ecological preferences have resulted in its unique physical characteristics, which include compact stems covered in pubescent hair, branches of different lengths (~ 55 cm), noticeable bracts (3–5 mm), and small leaves (11 mm-5-19)^9^. The distinctive attributes of *O. grossii* set it apart from other members of the Lamiaceae family, making it an intriguing subject for further investigation into its bioactive properties.

The *Thymus* genus, found in the Moroccan landscape, is notable for its significant diversity, boasting around nine endemic species. These plants possess a significant pharmacological value due to their ample presence of bioactive compounds, including phenolic acids, tannins, flavonoids, and resins. These compounds, which are present in both the aerial parts and essential oils, possess unique medicinal properties^6,10^. Notably, thymol and carvacrol are prominent constituents found in thyme oils. These compounds are recognized for their antioxidant, antiseptic, antibacterial, and antifungal properties^11^.

Within the domain of traditional healing, *T. pallidus,* a crucial member of the *Thymus* genus, assumes a prominent role. The historical use of this substance has included a variety of applications, such as powders and infusions, for the purpose of alleviating gastrointestinal disorders, whooping cough, bronchitis, influenza, and oral infections. The ethnobotanical uses mentioned are in accordance with scientifically supported biological activities, including antibacterial, antifungal, antioxidant, anti-inflammatory, analgesic, and spasmolytic effects^12^. The distinctive characteristics of *T. pallidus* highlight its therapeutic potential and align with the well-established reputation of the Lamiaceae family as a source of medicinal compounds.

Antimicrobial resistance (AMR) is a noteworthy issue and poses a significant threat to public health. While several mechanisms including genetic mutations and the acquisition of resistance genes through horizontal gene transfer contribute to the development of AMR, the misuse and overuse of antimicrobial drugs, both in human healthcare and in agriculture, contribute to the development and spread of the resistance^13^. The effect of AMR has hampered the treatment of infectious diseases, hence leading to increased morbidity, mortality, and healthcare costs. Notably, AMR continues to rise globally, hence, the search for a newer class of antimicrobial drugs remains unabated^14^. The phytochemical constituents of aromatic and medicinal plants have been to possess antimicrobial properties, with their EOs also possessing the ability to regulate pathogenic bacteria^15,16^. Within this particular context, it is imperative to undertake a comprehensive examination of the intricate tapestry presented by the various members of the Lamiaceae family, namely *O. grossii* and *T. pallidus.* This exploration aims to unravel the unique bioactive characteristics exhibited by these species and their significance within the realms of both traditional and contemporary medicinal practices.

This study has a dual objective: to showcase the value of *O. grossii* and *T. pallidus* plants and to explore their potential in combating radicals and bacteria. Specifically, the research aims to evaluate the antimicrobial properties of essential oils (EOs) extracted from these plants. The primary focus involves testing the effectiveness of these EOs against four bacterial strains—*Salmonella sp*, *Streptococcus sp*, *S. aureus*, and *E. coli*. These strains were isolated from patients' vomit or fecal samples collected at the emergency department of the University Hospital of Fez in Morocco. Through this assessment, the researchers intend to determine the presence of significant antimicrobial and antioxidant activity within these essential oils. The study's findings could shed light on the potential of these EOs as potent agents with antimicrobial and antioxidative properties, offering insights into natural solutions for these challenges.

## Materials and methods

### Plant material

Fresh leaves of *T. pallidus* and *O. grossii* were systematically collected in June 2017 from the region of Ribat EL Kheir located approximately 75 km from the city of Fez, Morocco (33° 49′ north, 4° 25′ west). The identification of the plant specimens was performed by Professor Amina Bari, a botanist affiliated with the Department of Biological Sciences in the Faculty of Science at Sidi Mohammed Ben Abdellah University, Fez, Morocco. Notably, *O. grossii* is given voucher number A51/08/06/2018/SE, whilst *T. pallidus* is given A52/08/06/2018/SE. The collected leaves were air-dried under suitable shade conditions to preserve their phytochemical composition. Subsequently, the dried leaf samples were stored in a dry and cool environment at a temperature of 5 °C until they were ready for further analysis or experimentation.

### Isolation of essential oils

Under the established protocols outlined in the European Pharmacopoeia^17^, the EOs were extracted from the air-dried plant materials using a Clevenger-type apparatus for 3 h. The EOs were collected in a sealed vial and subsequently stored at a temperature of 4 °C to ensure their preservation and maintain their quality. The yield of essential oil was determined using the formula:

1 $$\mathrm{Yield }\left(\mathrm{\%}\right)=\left(\frac{weight\,\, of \,\,obtained\,\, EO}{weight \,\,of \,\,the \,\,material \,\,plant}\right)\times 100,$$

where the weight of the obtained essential oil refers to the mass of essential oil extracted from the plant material and the weight of the plant material is the initial mass of the plant material used for extraction.

### Phytochemical analysis

The analysis of the extracted essential oils was carried out using gas chromatography-mass spectrometry (GC–MS) techniques. The analysis employed fused silica capillary columns, specifically SPB-1 and SupelcoWax-10, as described by reference^18^. These column types are commonly used for the separation and identification of volatile compounds present in EO^17^.

### Total flavonoids content (TFC) and the total phenolic content (TPC)

The aluminium chloride colorimetric assay was employed to assess the total flavonoid concentration of EOs as described by reference^19^. The solution was prepared, and the absorbance at 510 nm was measured using a Jasco v-530 spectrophotometer, with a blank sample for comparison. Galic acid was used as the standard to create a calibration curve. The overall quantity of flavonoid content in each sample was expressed in terms of gallic acid equivalents per gram of mg of EO (mg GAE/mg EO).

For the determination of the phenolic content in the samples, the Folin-Ciocalteu method was employed, as outlined by^20^. The reaction took place at room temperature in the dark for 2 h, and the absorbance at 760 nm was measured using a Jasco v-530 spectrophotometer. Gallic acid was used as the standard for comparison. The total phenol content was expressed in milligrams of gallic acid equivalents per gram of EO (mg GAE/mg EO). Each specimen was evaluated in triplicate to ensure the accuracy and reproducibility of the results.

### Antioxidant activity

#### DPPH radical scavenging activity

The DPPH radical-scavenging ability of the essential oils was assessed using the approach described by^21^. Noteworthy, this technique was originally developed by Blois in 1958^22^. After a 30-min incubation period at room temperature in the absence of light, the absorbance of the mixture was measured at 517 nm using a Jasco V-530 spectrophotometer. The percentage inhibition was calculated using the following formula:

2 $$\mathrm{I }\left(\mathrm{\%}\right)=\left(1-\frac{As}{A0}\right)\times 100 ,$$

where I (%): inhibition percentage, as sample absorbance, and A0: represents the absorbance of the blank control.

While BHT was utilized as a positive control in the experiment. The IC_50_ values were determined based on the concentration of the essential oils that inhibited the DPPH radical by 50%^23^.

#### Reducing power capacity

The assessment of reduction power for EO was conducted in accordance with the methodology outlined by^24^. Quercetin is utilized as the standard. The IC_50_ values, denoting the concentration at which the absorbance reached 0.5, were determined to quantitatively evaluate the reduction power. To achieve this, a graph was constructed by plotting the absorbance against the corresponding concentration, allowing for the determination of the IC_50_ value in milligrams per milliliter (mg/mL).

#### Total antioxidant capacity (TAC test)

The determination of the total antioxidant capacity of the EOs was performed as described by^25^. The assay was based on the conversion of Mo (VI) to Mo (V) and the subsequent formation of a green phosphate/Mo (V) complex under acidic conditions. The absorbance of the resulting complex was measured at 695 nm using a Jasco v-530 spectrophotometer. The antioxidant activity was expressed in terms of ascorbic acid equivalents (mg AAE/g DW), providing a quantitative measure of the overall antioxidant capacity.

### Antibacterial activity

#### Bacterial strains

In this study, the antibacterial activity of oregano and thyme EO against four bacterial strains, including Gram-positive Staphylococcus aureus and Streptococcus faecalis D, as well as Gram-negative Escherichia coli and Salmonella, provided by the Regional Laboratory of Epidemiological Diagnosis and Environmental Hygiene in Fez, Morocco. The density was changed to correspond to a 0.5 McFarland Standard’s turbidity, equivalent to 1–5 108 CFU per milliliter. Agar disc diffusion analysis^26^.

#### Agar well diffusion (AWD) assay

The AWD test was run in triplicate using a modified version of the Kirby-Bauer method^27^ Standardized suspensions (108 CFU/mL) were used to inoculate Mueller Hinton agar plates before Whatman paper discs (3 mm) were applied to the agar's surface. After that, essential oils were infused into the discs. At 37 °C, all plates were incubated for a day. The widths of the inhibitory zones were measured after incubation using a ruler. By assessing the zones of inhibition against the examined bacterial strains, the antibacterial efficacy was evaluated.

#### Minimum inhibitory concentration (MIC)

With some modifications, the National Committee for Clinical Laboratory Standards' experiment^28^ was used to determine the MIC and 96-well plates were used for the test. The different concentrations of oregano EOs and antibiotics were prepared in a suspension containing 0.2% agar in sterile distilled water^29^, they were carried out using successive 1/2 dilutions of the EOs, ranging from 5000 to 9 µg/mL, and the antibiotics, ranging from 200 to 0.4 µg/mL. The corresponding well of the plate was filled with varying amounts of antibiotics or oregano EO. The concentrations obtained in the wells ranged from 1250 to 2 μg/mL. The presence of bacteria was determined by adding 20 μl of a 10% aqueous solution of 2.3.5-triphenyl tetrazolium chloride to each well. The lowest concentration that doesn’t result in a red hue was designated as MIC^26,30^.

### Molecular docking

The molecular docking methodology utilized a virtual screening process to analyze the crystal structures of different antibacterial and antioxidant proteins from the RCSB protein data bank, specifically PDB IDs 1AJ6^31^ and 6QME^32^. The decision to select the crystal structures of these proteins for molecular docking methodology was likely driven by their alignment with the study's focus on assessing the antimicrobial and antioxidant activities of essential oils. These protein targets have been chosen based on their relevance to the study’s objectives, potential roles in antibacterial^33–35^ and antioxidant^36–38^ processes as indicated by prior research or literature, structural availability in the RCSB Protein Data Bank, potential for validation and benchmarking, and the feasibility of computational analysis^39–44^. Such protein selection allows for investigating interactions with known proteins that are pertinent to the study’s goals, contributing to a comprehensive understanding of the essential oils’ potential effects. To perform this analysis, various software tools were employed, including MGLtools, Autodock4, Autogrid4^45^, BIOVIA Discovery Studio Visualizer^46^, Chemdraw Ultra^47^, and Chemdraw 3D Pro^48^.

Initially, the protein structures were processed using BIOVIA’s Discovery Studio Visualizer, wherein heteroatoms, co-crystal ligands, and solvent molecules were eliminated. To optimize the protein structure for docking, Autodock tools were utilized, assigning appropriate polar hydrogen and Kollman charges. The resulting optimized protein structure was saved as a pdbqt file^49^. For the ligands, ChemDraw Ultra was used to draw their structures, followed by energy minimization in Chem 3D Pro. The ligands were then converted to the pdbqt file format using the OpenBabel GUI program^50^. The structure-based virtual screening was conducted using Autodock4, with each ligand drug docked independently into the active site of each protein. The interactions between the ligands and proteins were visualized using BIOVIA Discovery Studio Visualizer. To validate the results, the root-mean-square deviation (RMSD) value was calculated, and the co-crystal ligand was re-docked. Poses were accepted if both the docking and experimental ligand RMSD values were less than 2.0^51,52^.

### Statistical analysis

To analyze the obtained data, the means of the three-way analyses were calculated, and the results were presented as mean ± standard deviation (SD). The IBM SPSS Statistics software version 20.0 was utilized to perform the statistical analysis. The Fisher’s least significant difference (LSD) test and one-way analysis of variance (ANOVA) were employed to assess the statistical significance between different groups at a significance level of *P* ≤ *0.05*.

### Plant collection approval

No approval is needed to collect *Origanum grossii* and *Thymus pallidus* in Morocco for research purposes.

### IUCN policy statement

The collection of plant material complies with relevant institutional, national, and international guidelines and legislation.

## Results

### Chemical composition of EOs

The extraction of essential oils (EOs) from the leaves of *O. grossii* and *T. pallidus* resulted in yields of 3% and 1% (v/w), respectively, on a dry weight basis. The extracted EOs were then subjected to gas chromatography-mass spectrometry (GC–MS) analysis, and the results are presented in Tables 1 and 2.

**Table 1:** The phytochemical constituents of the EOs from *T. pallidus*.

RT	Chemical names	RI		Chemical formula	Chemical class	Area (%)
		Obs	Lit			
15.58	*p*-cymene	1022	1024	C_10_H_14_	Monoterpene hydrocarbons (MT.H)	0.563
17.96	Santolina triene	906	908	C_10_H_16_	MT.H	2.036
16.64	ɤ-terpinene	1055	1059	C_10_H_16_	MT.H	0.632
17	Sabinene hydrate	1067	1070	C_10_H_18_O	Monoterpene oxygenated (MT.O)	0.231
17.64	Linalool oxide	1070	1072	C_10_H_18_O	MT.O	0.146
20.23	Borneol	1168	1169	C_10_H_18_O	MT.O	0.986
20.90	Terpinen-4-ol	1174	1177	C_10_H_18_O	MT.O	0.741
24.03	Thymol	1288	1290	C_10_H_14_O	MT.O	86.354
27.31	Caryophyllene	1418	1419	C_15_H_24_	Sesquiterpene hydrocarbons (ST.H)	0.324
29.23	Himachalene	1450	1451	C_15_H_24_	ST.H	1.631
31.56	Caryophyllene oxide	1563	1667	C_15_H_24_O	Sesquiterpene oxygenated (ST.O)	0.463
32.22	*Trans*-cadinol	1639	1640	C_15_H_26_O	ST.O	0.128
			Monoterpene hydrocarbons (MT.H)			3.231
			Monoterpene oxygenated (MT.O)			88.458
			Sesquiterpene hydrocarbons (ST.H)			1.955
			Sesquiterpene oxygenated (ST.O)			0.591
			Total (%)			94.235

*RI* retention indices, *RT* retention time in minutes, *Obs* retention indices calculate, *Lit* literature.

**Table 2:** The phytochemical constituents of the EOs from *O. grossii*.

RT	Chemical names	RI		Chemical formula	Chemical class	Area (%)
		Obs	Lit			
15.56	*p*-cymene	1020	1024	C_10_H_14_	Monoterpene hydrocarbons (MT.H)	0.364
16.62	ɤ-terpinene	1054	1059	C_10_H_16_	MT.H	0.423
18.59	*Cis*-sabinene	1070	1070	C_10_H_16_	MT.H	1.063
15.78	Eucalyptol	1029	1031	C_10_H_18_O	Monoterpene oxygenated (MT.O)	0.125
19.56	α-campholenal	1124	1126	C_10_H_16_O	MT.O	1.157
20.22	Isoborneol	1160	1160	C_10_H_18_O	MT.O	0.983
20.47	Borneol	1168	1169	C_10_H_18_O	MT.O	0.897
21.34	isopulegone	1592	1596	C_10_H_16_O	MT.O	0.143
23.05	Myrtenyl acetate	1321	1326	C_12_H_18_O_2_	Other (O)	0.684
24.56	Carvacrol	1296	1299	C_10_H_14_O	MT.O	70.963
27.33	Caryophyllene	1418	1419	C_15_H_24_	Sesquiterpene hydrocarbons (ST.H)	2.376
29.23	ɤ- Maaliene	1472	1477	C_15_H_24_	ST.H	0.637
27.82	Valencene	1494	1494	C_15_H_24_	ST.H	0.218
31.55	Caryophyllene oxide	164	1667	C_15_H_24_O	Sesquiterpene oxygenated (ST.O)	0.921
			Monoterpene hydrocarbons (MT.H)			1.850
			Monoterpene oxygenated (MT.O)			74.268
			Sesquiterpene hydrocarbons (ST.H)			3.231
			Sesquiterpene oxygenated (ST.O)			0.921
			Other (O)			0.684
			Total (%)			80.954

*RI* retention indices, *RT* retention time in minutes, *Obs* retention indices calculate, *Lit*. literature.

The GC–MS analysis provided in Table 1 showing detailed insights into the chemical constituents of the Eos from *T. pallidus*. Key compounds, including p-cymene, γ-terpinene, Sabinene hydrate, linalool oxide, Santolina triene, Borneol, Terpinen-4-ol, Thymol, Caryophyllene, Himachalene, Caryophyllene oxide, and Trans-cadinol, were identified and quantified based on their retention indices, retention times, and respective areas in the chromatogram. Thymol, in particular, dominated the composition of the *T. pallidus* EO, constituting a significant proportion (86.354%) of the total.

Table 2 outlines the comprehensive profile of phytochemical constituents within the *O. grossii* EOs. Through GC–MS analysis, specific compounds such as p-cymene, Eucalyptol, γ-terpinene, Cis-sabinene, α-campholenol, Isoborneol, Borneol, Isopulegone, Myrtenyl acetate, Carvacrol, Caryophyllene, Valencene, and γ-Mamliene are identified and quantified. Notably, Carvacrol stands out as the predominant component, constituting a substantial proportion (70.963%) of the total composition. These details enrich our understanding of the chemical composition and potential bioactivity of the *O. grossii* EO.

The exploration of the phytochemical constituents within the essential oils (EOs) of *T. pallidus*, as conducted in this study, unveiled a total of 12 compounds, collectively encompassing 94.235% of the total composition. A noteworthy revelation is the prevalence of thymol, which emerged as the principal compound, constituting a substantial majority at 86% (Table 1).

In contrast, the investigation into the phytochemical makeup of *O. grossii* EO disclosed the presence of 14 distinct compounds that set it apart from *T. pallidus*. Notably, Carvacrol stood out as the predominant constituent, accounting for a significant proportion of 70% within the EOs (Table 2).

### Antioxidant activity

#### DPPH scavenging activity

The antioxidant activity of the EOs was evaluated using the DPPH scavenging activity assay which is a frequently utilized method for antioxidant activity evaluation. The results presented in Table 3 elucidate the DPPH radical-scavenging potential of *O. grossii* and *T. pallidus* EOs. Notably, the EOs of *O. grossii* exhibited the highest radical-scavenging ability, as evident by its IC_50_ value of 0.073 ± 0.001 mg/mL, followed by *T. pallidus* EO with an IC_50_ value of 0.131 ± 0.002 mg/mL. Interestingly, statistical analysis revealed that *O. grossii* EO displayed significantly stronger antioxidant activity (*P* ≤ *0.05*) in comparison to the pure reference antioxidant BHT, with an IC_50_ value of 0.120 ± 0.001 mg/mL.

**Table 3:** DPPH radical scavenging activity and Ferric reducing/antioxidant power (FRAP) capacity.

	*O. grossii*	*T. pallidus*	BHT	Quercetin
IC_50 (_mg/mL_)_	0.073 ± 0.001***	0.131 ± 0.002	0.120 ± 0.001	–
EC_50 (_mg/mL_)_	56.333 ± 1.778***	79.333 ± 1.556***	–	33.450 ± 0.027

IC_50_: half maximal inhibitory concentration, EC_50_: half maximal effective concentration, data are reported as mean values ± SD of three measurements. Means were significantly different when ***P ≤ *0.001.*

#### Ferric reducing power (FRAP)

As depicted in Table 3, the EOs of *O. grossii* exhibited superior reducing power against ferric ions compared to the EOs *of T. pallidus*, yielding values of 56.333 ± 1.778mg/mL and 79.333 ± 1.556mg/mL.

#### Total antioxidant activity (TAC)

**Figure 1:**
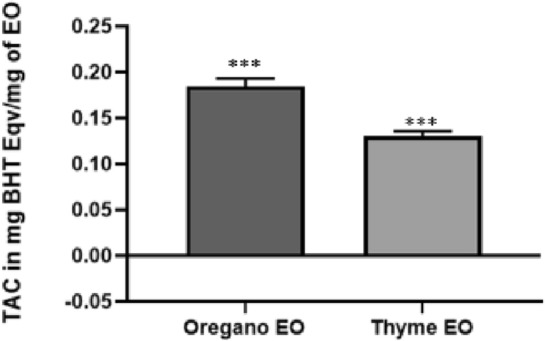
The total antioxidant capacity of *O. grossii and T. pallidus* Eos.

The results, presented in Fig. 1, were quantified in terms of BHT equivalents (mg BHT E/mg EO). Significantly higher TAC values were observed for the EOs pf *O. grossii* compared to *T. pallidus*, with recorded values of 0.185 ± 0.005 mg BHT E/mg EO and 0.13 ± 0.004 mg BHT E/mg EO, respectively.

### Quantification of total flavonoid content (TFC) and total phenolic content (TPC)

The determination of TFC and TPC for each test sample is elaborated in Table 4. The TFC values were standardized with respect to the reference quercetin, as detailed in the same table. In a similar manner, the TPC outcomes were aligned with the benchmark gallic acid and were expressed as milligrams of gallic acid equivalents per gram of the sample (mg GAE/g of the sample). Noteworthy is the observation that among the samples, *O. grossii* demonstrated the most substantial TFC and TPC values, exhibiting measurements of 0.207 ± 0.007 mg QE/g EO and 136.66 ± 0.003 mg GAE/g EO, respectively.

**Table 4:** Total phenolic (TPC) and total flavonoids (TF) contents.

	*O. grossii*	*T. pallidus*
Flavonoids in mg QE/mg EO	0.207 ± 0.007^a^	0.109 ± 0.006^b^
Polyphenols in mg GAE/mg EO	0.136 ± 0.003^a^	0.125 ± 0.002^b^

Data are reported as mean values ± SD of three measurements. Means were significantly different when P < 0.05; values followed by different letters are significantly different.

### Pearson’s correlation analysis

In order to explore the interdependence between the total phenolic and flavonoid contents and the observed antioxidant activity, Pearson’s correlation coefficient analysis was employed in this study, as delineated in Table 5. The outcome of this analysis unveiled noteworthy and statistically significant correlations among these variables.

**Table 5:** Pearson correlation between antioxidant activity parameters.

	Pearson correlation				
	IC_50_	EC_50_	TAC	Flavonoids	Polyphenols
IC_50_	1				
EC_50_	0.183	1			
TAC	− 0.981**	− 0.969**	1		
Flavonoids	− 0.981**	− 0.997**	0.977**	1	
Polyphenols	− 0.852*	− 0.780	0.871*	0.814*	1

Correlations are: *significant at p < 0.01, **significant at p < 0.001.

### Antibacterial activity

**Figure 2:**
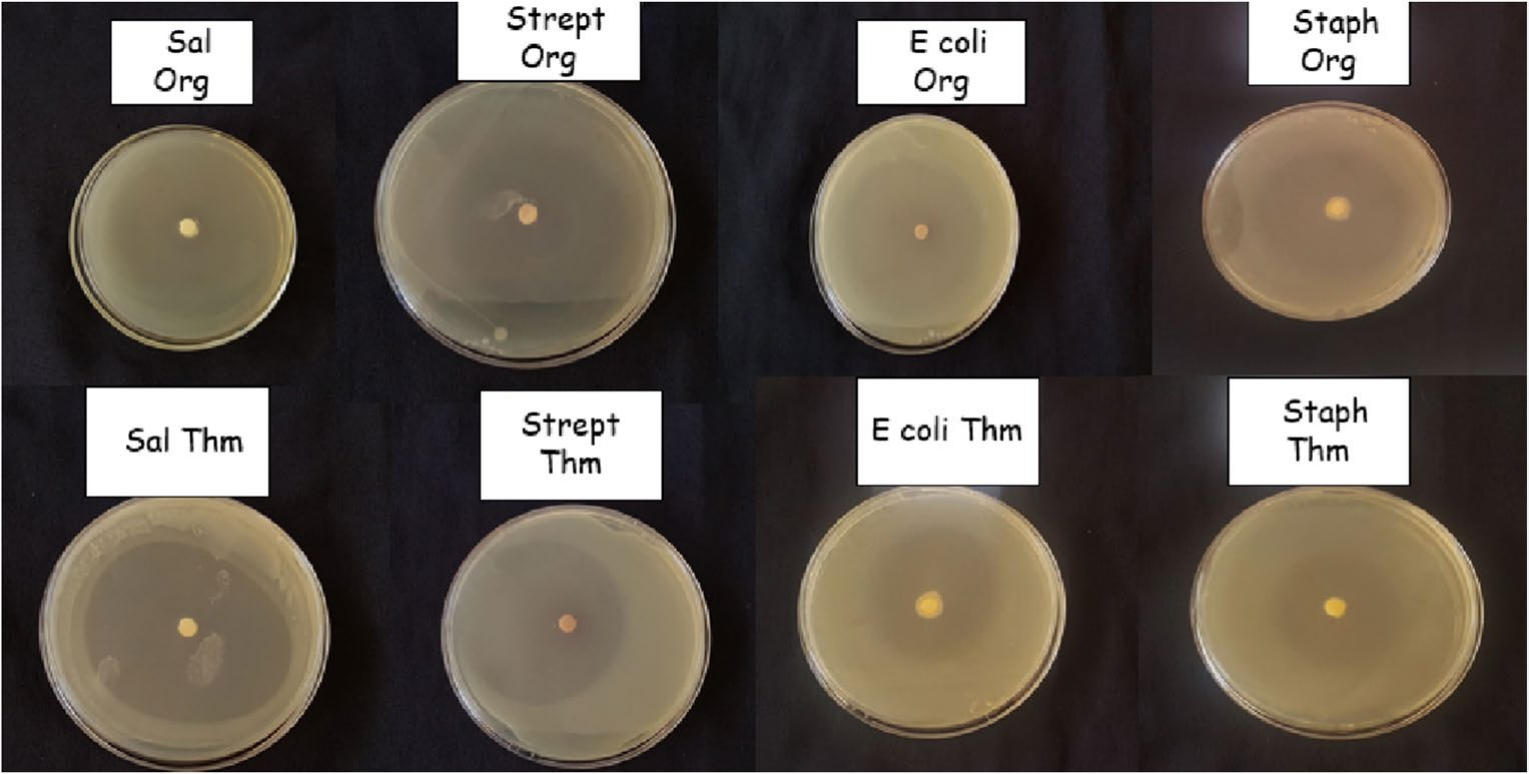
Antibacterial activity of *O. grossii* and *T. pallidus* EOs.

The outcomes of the agar disc diffusion antibacterial activity test are detailed in Table 6 and illustrated in Fig. 2. The results indicate that both essential oils (EOs) demonstrated significant antibacterial effects against all bacterial strains tested. Notably, the EOs exhibited heightened efficacy against a spectrum encompassing both Gram-positive and Gram-negative bacteria. Specifically, noteworthy antibacterial activity was observed against Salmonella sp., with *O. grossii* and *T. pallidus* EOs yielding the most substantial inhibition zone diameters of 42.6 ± 0.88 mm and 36 ± 0.5 mm, respectively. It is worth noting that the efficacy of *O. grossii* EO and *T. pallidus* EO against *E. coli* was comparatively lower.

**Table 6:** Antibacterial activity of *Origanum grossii*, *Thymus pallidus*, and some antibiotics.

Bacteria	Essential oils				Antibiotics							
	*O grossii*		*T pallidus*		OX_5_	S_25_	CRO_30_	ZOX_30_	OFX_5_	CN_15_	P_10_	CEC_30_
	IZ	CMI	IZ	CMI	IZ	IZ	IZ	IZ	IZ	IZ	IZ	IZ
*Staph. aureus*	30.67 ± 0.89	0.61	26.67 ± 0.45	2.44	0	12	20	11	14	20	0	0
*Strep. fecalus*	32.33 ± 0.45	0.61	30.00 ± 0.67	0.61	0	14	0	0	27	14	0	0
*Salmonella sp*	42.67 ± 0.88	0.31	35.33 ± 0.45	0.31	0	16	18	12	20	20	0	0
*E. coli*	33.00 ± 2.00	0.61	28.33 ± 1.11	0.61	0	14	22	13	14	21	0	0

*IZ* inhibition zone(mm), *CMI* minimum inhibitory concentration(ug/mL), *OX5* Oxacillin, *S25* streptomycin, *CRO30* ceftriaxone, *ZOX30* ceftizoxime, *OFX5* ofloxacin, *CN15* gentamicin, *P10* penicillin G, *CEC30* cefaclor.

When contrasting the antibacterial activity of the essential oils (EOs) with that of conventional antibiotics employed as positive controls, it becomes evident that the EOs displayed heightened potency. Through the microdilution technique, the efficacious concentrations of *O. grossii* and *T. pallidus* EOs were ascertained to fall within the range of 0.31 to 2.44 µg/mL, as outlined in Table 6. Particularly noteworthy is the concentration of 0.31 µg/mL, which emerged as the most efficacious against *Salmonella sp.* for both EOs.

### Molecular docking

The two macromolecules i.e. antibacterial protein PDB ID: 1AJ6 and antioxidant proteins 6QME were docked with the various types of phytochemical constituents of essential oils obtained from the plants *T. pallidus* and *O. grossii* and the results of docking were tabulated in the form of tables.

**Figure 3:**
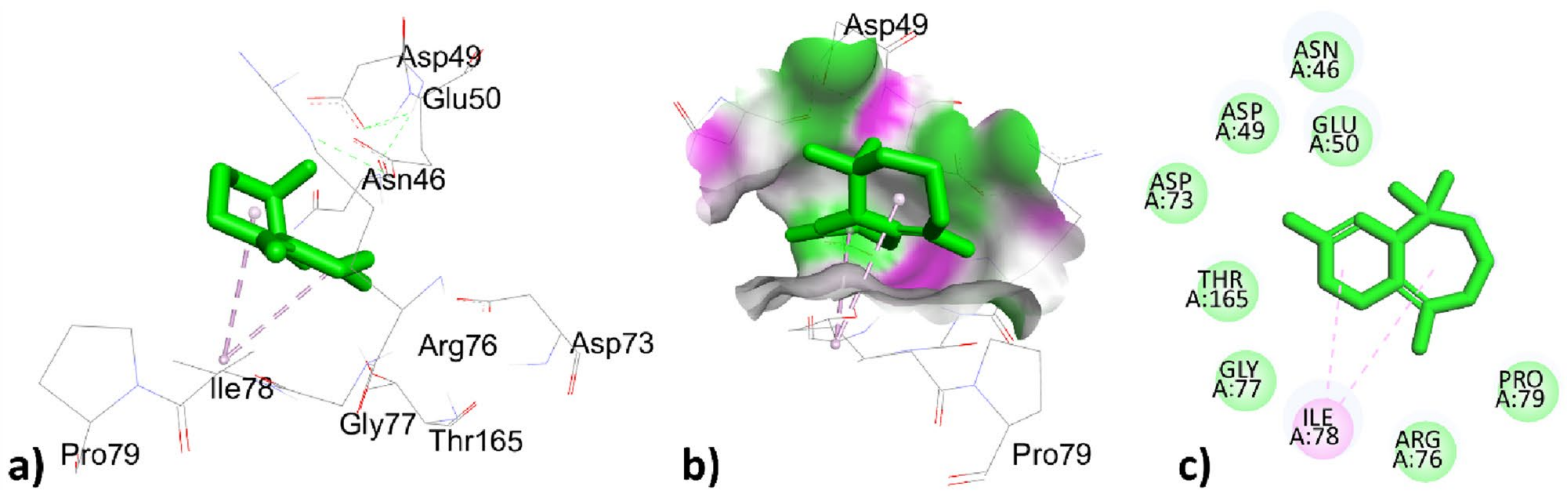
3D, hydrogen surface and 2D interaction of himachalene with 1AJ6.

**Figure 4:**
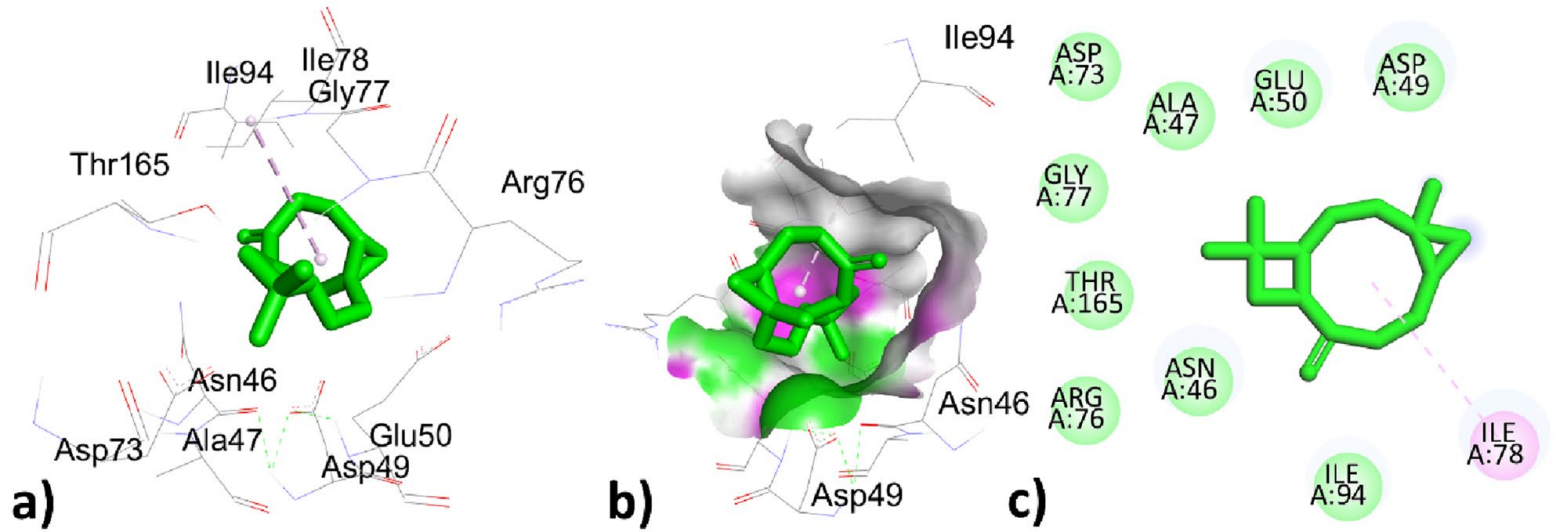
3D, hydrogen surface and 2D interaction of caryophyllene oxide with 1AJ6.

The phytochemical constituents p-cymene, santolina triene, ɤ-terpinene, sabinene hydrate, linalool oxide, borneol, terpinen-4-ol, thymol, caryophyllene, himachalene, caryophyllene oxide, trans-cadinol was extracted from *T. pallidus* plants virtually docked with antibacterial 1AJ6 proteins and showed binding affinity of − 5.7, − 4.9, − 5.7, − 5.5, − 5.0, − 4.8, − 5.5, − 4.8, − 5.5, − 5.7, − 5.9, − 6.7, − 6.0, − 5.5 in (Kcal/mol) respectively tabulated in Table 7. The p-cymene showed hydrophobic interaction with amino acids VAL43, VAL120, VAL167, and ILE78 with distances 4.169, 3.982, 4.336, 5.287 (Å), santolina triene was bounded hydrophobically with distances 4.092, 5.232, 5.279 (Å) to amino acid residues ALA47, ILE78, VAL43, ɤ-terpinene showed two types of interaction with 1AJ6, one was electrostatic interaction to amino acid residues ASP49 with bond angle 4.092 (Å) and the other was hydrophobic interaction to amino acid ILE78 with bond distance 5.272 (Å). Sabinene hydrate hydrophobically interacted with amino acid residues ALA47, ILE78, and VAL43 with bond distances 4.092, 5.231, 5.279 (Å), linalool oxide showed two types of interactions, one with GLU50 was bounded through a hydrogen bond with bond distance 2.668 (Å) and ILE78, ILE94 interacted hydrophobically with bond distance 3.745, 3.634. In the Borneol-1AJ6 docking results, amino acid ILE78 was bounded through hydrophobic interaction with bond distance 5.026 (Å) and amino acids GLU50, and ARG76 were bounded through a hydrogen bond with bond distance 2.048, 2.7565 (Å) mentioned in Table 7. The Terpinen-4-ol showed hydrophobic interaction with distances 5.156, 3.772, 4.467(Å) to amino acid residues ILE78, VAL120, and VAL167 showed in. Thymol interacted hydrophobically with amino acids VAL167, ILE78 with bond angles 4.702, 5.038 (Å) and interacted through hydrogen bond to amino acids residues ASP73, ALA47 with bond distances 2.003, 3.473 (Å) tabulated in Table 7. Caryophyllene interacted hydrophobically with residues ARG190, and PHE41 of 1AJ6 with bond distances 5.293, 4.599 (Å); *Trans*-cadinol interacted hydrophobically with amino acids residues ARG190, LYS189, and ARG190, PHE41 with bond distance 4.997, 3.991, 4.076, 5.137 (Å) tabulated in Table 7. The highest top-ranked docked essential oil himachalene concerning its binding affinity -6.7 (Kcal/mol) has interacted through hydrophobic interaction with amino acids ILE78 with bond distances 4.977, 4.849 (Å) represented in Fig. 3 and Table 7. The second highest essential oil caryophyllene oxide with binding energy − 6.0 (Kcal/mole) bounded hydrophobically to ILE78 with a bond distance of 5.150 (Å) represented in Fig. 4 and Table 7. The two compounds himachalene and caryophyllene oxide have the highest binding score with antibacterial protein 1AJ6 thus they were considered good antibacterial targets.

**Table 7:** Active site interactions (type of interactions, bond distance in Å) of phytochemical constituents of *T. pallidus* plant with antibacterial protein 1AJ6 along with docking score (Kcal/mol).

Interaction of EOs from *T. pallidus* with 1AJ6	Residues	Types of interaction	Bond distance (Å)	Binding affinity (Kcal/mol)
*p*-cymene	VAL43	Hydrophobic	4.169	− 5.7
	VAL120	Hydrophobic	3.982	
	VAL167	Hydrophobic	4.336	
	ILE78	Hydrophobic	5.287	
Santolina triene	ALA47	Hydrophobic	4.092	− 4.9
	ILE78	Hydrophobic	5.232	
	VAL43	Hydrophobic	5.279	
ɤ-terpinene	ASP49	Electrostatic	4.974	− 5.7
	ILE78	Hydrophobic	5.272	
Sabinene hydrate	ALA47	Hydrophobic	4.092	− 5.5
	ILE78	Hydrophobic	5.231	
	VAL43	Hydrophobic	5.279	
Linalool oxide	GLU50	Hydrogen bond	2.668	− 5.0
	ILE78	Hydrophobic	3.745	
	ILE94	Hydrophobic	3.634	
Borneol	GLU50	Hydrogen bond	2.048	− 4.8
	ARG76	Hydrogen bond	2.756	
	ILE78	Hydrophobic	5.026	
Terpinen-4-ol	ILE78	Hydrophobic	5.156	− 5.5
	VAL120	Hydrophobic	3.772	
	VAL167	Hydrophobic	4.467	
Thymol	ASP73	Hydrogen bond	2.003	− 5.7
	ALA47	Hydrogen bond	3.473	
	VAL167	Hydrophobic	4.702	
	ILE78	Hydrophobic	5.038	
Caryophyllene	ARG190	Hydrophobic	5.293	− 5.9
	PHE41	Hydrophobic	4.599	
Himachalene	ILE78	Hydrophobic	4.977	− 6.7
	ILE78	Hydrophobic	4.849	
Caryophyllene oxide	ILE78	Hydrophobic	5.150	− 6.0
*Trans*-cadinol	ARG190	Hydrophobic	4.997	− 5.5
	LYS189	Hydrophobic	3.991	
	ARG190	Hydrophobic	4.076	
	PHE41	Hydrophobic	5.137

In this study Table 8 represents interaction of antibacterial protein 1AJ6 phytochemical constituents *p*-cymene, ɤ-terpinene, *Cis*-sabinene, Eucalyptol, α-campholenal, isoborneol, borneol, isopulegone, myrtenyl acetate, carvacrol, caryophyllene, ɤ-maaliene, valencene, caryophyllene oxide of essential oils obtained from *O. grossii* and co-crystallized ligand novobiocin with their binding score − *5.7, *− *5.7, *− *4.9, *− *4.9, *− *4.7, *− *4.7, *− *4.8, *− *5.3, *− *5.6, *− *6.1, *− *5.9, *− *6.4, *− *7.3, *− *6.0, *− *7.6* (Kcal/mol) respectively showed in Table 8. Some of the constituents of *O. grossii* such as *p-cymeme,* ɤ-terpinene, borneol, caryophyllene, caryophyllene oxide were the same as with the constituents of *T. pallidus* already described above and tabulated in Tables 7 and 8. The remaining constituents like *cis*-sabinene were hydrophobically bound to amino acid residues of 1AJ6 ILE78, ILE78, and ILE94 with distances 4.349, 3.872, 3.776 (Å); the eucalyptol was bounded hydrophobically to ILE94, ALA100 with bond distance 5.397, 4.991 (Å); α-campholenal, isoborneol and caryophyllene oxide were interacted hydrophobically to ILE78 with bond distance 4.529, 4.878, 5.150 (Å) respectively tabulated in Table 8. The isopulegone has interacted hydrophobically with amino acids VAL120, and VAL167 with distances 4.112, and 4.401 (Å); myrtenyl acetate showed two types of interaction one was hydrophobic to ILE78 with a distance of 2.251 (Å), and one was hydrogen bond interaction to THR165 with distance 5.302 (Å); Carvacrol has interacted through a hydrogen bond to ASP73, ALA47 and hydrophobic bond to ILE78 with bond distance 2.126, 3.562, 5.203 respectively in Table 8.

**Table 8:** Active site interactions (type of interactions, bond distance in Å) of phytochemical constituents of *O. grossii* plant with antibacterial protein 1AJ6 along with docking score (Kcal/mol).

Interaction of EOs from *O. grossii* with 1AJ6	Residues	Types of interaction	Bond distance (Å)	Binding affinity (Kcal/mole)
*p*-cymene	VAL43	Hydrophobic	4.169	− 5.7
	VAL120	Hydrophobic	3.982	
	VAL167	Hydrophobic	4.337	
	ILE78	Hydrophobic	5.287	
ɤ -terpinene	ASP49	Electrostatic	4.974	− 5.7
	ILE78	Hydrophobic	5.272	
*Cis*-sabinene	ILE78	Hydrophobic	4.349	− 4.9
	ILE78	Hydrophobic	3.872	
	ILE94	Hydrophobic	3.776	
Eucalyptol	ILE94	Hydrophobic	5.397	− 4.9
	ALA100	Hydrophobic	4.991	
α-campholenal	ILE78	Hydrophobic	4.529	− 4.7
Isoborneol	ILE78	Hydrophobic	4.847	− 4.6
Borneol	GLU50	Hydrogen bond	2.048	− 4.8
	ARG76	Hydrogen bond	2.756	
	ILE78	Hydrophobic	5.026	
isopulegone	VAL120	Hydrophobic	4.112	− 5.3
	VAL167	Hydrophobic	4.401	
Myrtenyl acetate	THR165	Hydrogen bond	2.251	− 5.6
	ILE78	Hydrophobic	5.302	
Carvacrol	ASP73	Hydrogen bond	2.126	− 6.1
	ALA47	Hydrogen bond	3.562	
	ILE78	Hydrophobic	5.203	
Caryophyllene	ARG190	Hydrophobic	5.293	− 5.9
	PHE41	Hydrophobic	4.599	
ɤ -Maaliene	ILE78	Hydrophobic	4.594	− 6.4
	ILE78	Hydrophobic	4.522	
Valencene	ILE78	Hydrophobic	4.648	− 7.3
	ILE78	Hydrophobic	3.989	
	VAL43	Hydrophobic	4.628	
	VAL167	Hydrophobic	4.579	
Caryophyllene oxide	ILE78	Hydrophobic	5.150	− 6.0
co-crystallized ligand novobiocin	THR165	Hydrogen bond	2.167	− 7.6
	LYS103	Hydrogen bond	3.419	
	ASN46	Hydrogen bond	4.016	
	ILE78	Hydrophobic	3.937	
	GLY77	Hydrophobic	3.879	
	ILE78	Hydrophobic	4.324	
	ILE94	Hydrophobic	5.034	
	PRO79	Hydrophobic	4.994	

**Figure 5:**
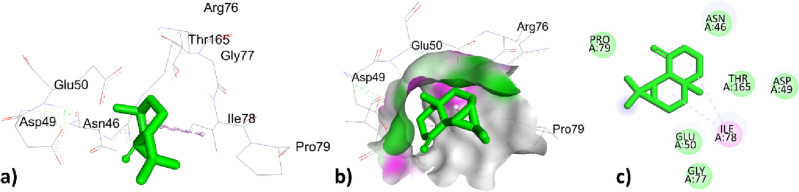
3D, hydrogen surface and 2D interaction of *ɤ* -Maaliene with 1AJ6.

**Figure 6:**
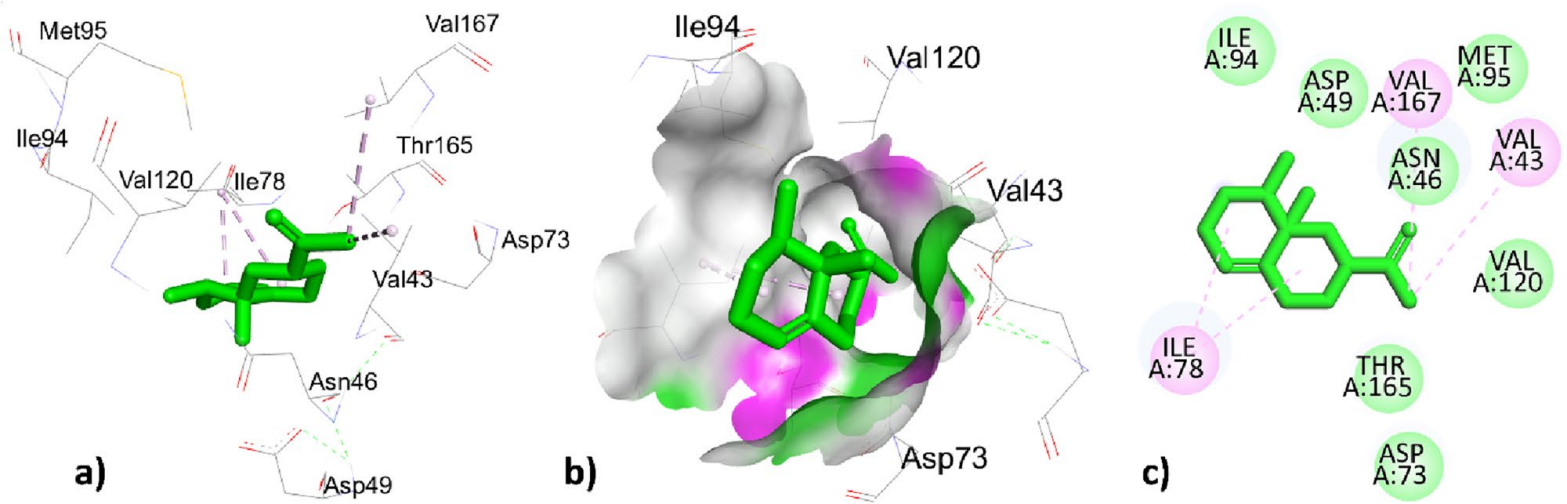
3D, hydrogen surface and 2D interaction of Valencene with 1AJ6.

The highest top ranked constitute valencene with a docking score of − 7.3 (Kcal/mol) has interacted through hydrophobic interaction with amino acids residues ILE78, ILE78, VAL43, and VAL167 with bond distances 4.648, 3.989, 4.628, 4.579 (Å) respectively represented in Fig. 5 and Table 8. The second highest top ranked ɤ- maaliene with a docking score of -6.4 (Kcal/mol) hydrophobically interacted with ILE78 with bond distances 4.594, 4.522 (Å) represented in Fig. 6 and Table 8.

**Figure 7:**
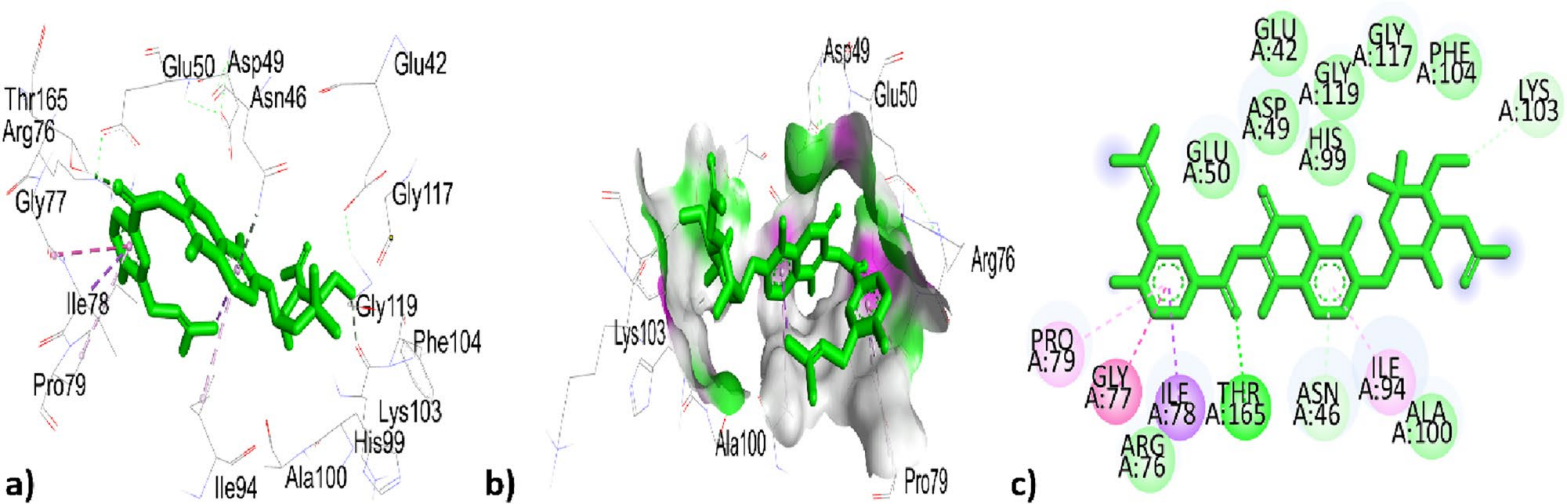
3D, hydrogen surface and 2D interaction of co-crystallized ligand novobiocin with 1AJ6.

The co-crystallized ligand novobiocin has greater binding affinity 0f. − 7.6 (Kcal/mol) with respect to all the phytochemical constituents of *T. pallidus* and *O. grossii* plants have interacted with two types of interactions; one was hydrogen bond interaction to residues THR165, LYS103, ASN46 with bond distance 2.167, 3.419, 4.016 (Å) and the second was hydrophobic interaction to ILE78, GLY77, ILE78, ILE94, PRO79 with bond distance 3.937, 3.879, 4.324, 5.034, 4.994 (Å) respectively represented in Fig. 7 and Table 8.

**Figure 8:**
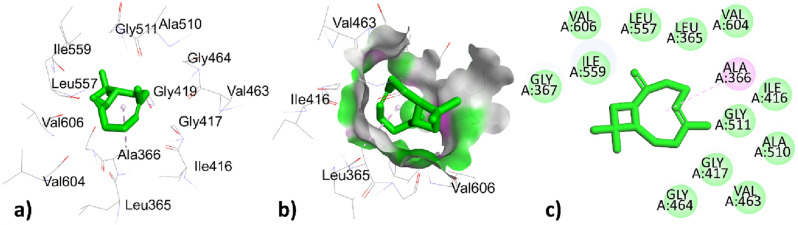
3D, hydrogen surface and 2D interaction of Caryophyllene with 6QME.

**Figure 9:**
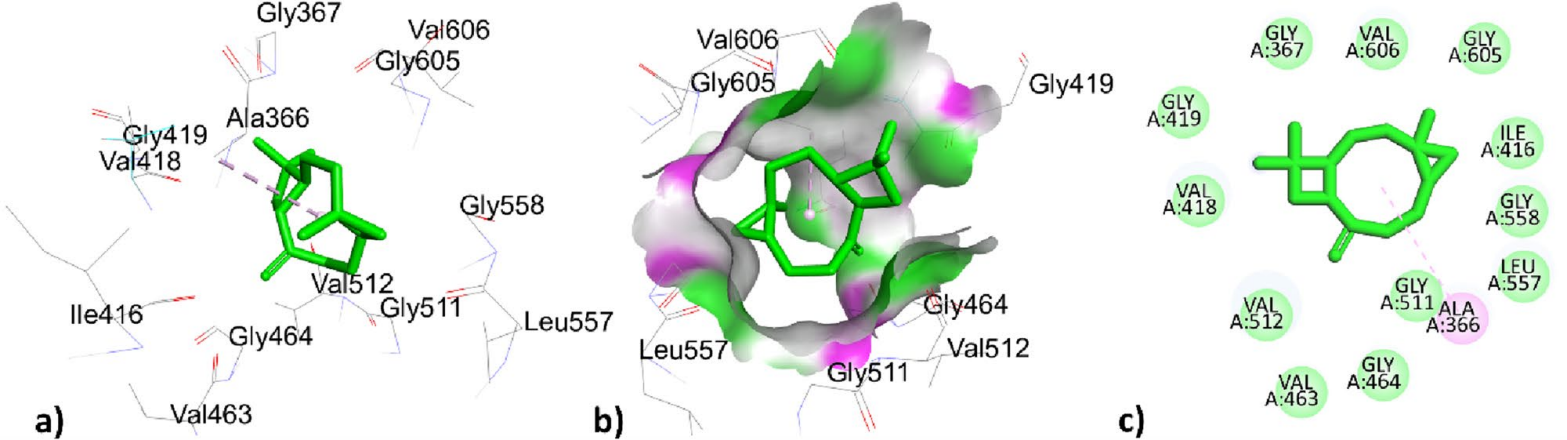
3D, hydrogen surface and 2D interaction of Caryophyllene oxide with 6QME.

Table 9 represented the interactions of phytochemical constituents p-cymene, santolina triene, ɤ-terpinene, sabinene hydrate, linalool oxide, borneol, terpinen-4-ol, thymol, caryophyllene, himachalene, caryophyllene oxide, trans-cadinol were extracted from *T. pallidus* plants virtually docked with antioxidant 6QME proteins and showed binding score of − 5.2, − 4.7, − 5.2, − 5.8, − 6.2, − 6.1, − 5.7, − 5.9, − 7.5, − 7.1, − 7.9, − 7.0 (Kcal/mol). As we have discussed earlier some of the constituents are same for the both plants such as *p-cymeme,* ɤ-terpinene, borneol, caryophyllene, caryophyllene oxide, the type of interaction with residues and binding affinity were the same after docked with the protein 6QME tabulated in Tables 9 and 10. The p-cymene hydrophobically interacted with ALA366 with a distance of 3.586 (Å); ɤ-terpinene interacted hydrophobically with TYR572, TYR334, TYR334, ALA556 with a distance of 3.625, 4.655, 4.338, 5.320 (Å); Borneol was bounded hydrophobically to ALA366 with bond length 4.791 (Å) while bounded through hydrogen bond interaction to ILE559 with distance 2.177 (Å) in Tables 8 and 9. The highest top-ranked compound Caryophyllene oxide with a binding affinity of − 7.9 and the 2nd highest top-ranked compound Caryophyllene with a binding affinity of − 7.5 hydrophobically interacted with ALA366 with a distance of 4.179, 4.941 (Å) tabulated in Tables 9 and 10 and interactions represented in Figs. 8 and 9

**Table 9:** Active site interacting residues, distance (Å) and binding affinity (Kcal/mol) of phytochemical constituents of *T. pallidus* with 6QME.

Interaction of EOs from *T. pallidus* with 6QME	Residues	Types of interaction	Bond distance (Å)	Binding affinity (Kcal/mole)
*p*-cymene	ALA366	Hydrophobic	3.586	− 5.2
Santolina triene	ALA366	Hydrophobic	3.915	− 4.7
	VAL465	Hydrophobic	4.314	
ɤ-terpinene	TYR572	Hydrophobic	3.625	− 5.2
	TYR334	Hydrophobic	4.655	
	TYR334	Hydrophobic	4.338	
	ALA556	Hydrophobic	5.320	
Sabinene hydrate	ALA366	Hydrophobic	3.915	− 5.8
	VAL465	Hydrophobic	4.314	
Linalool oxide	ILE416	Hydrogen bond	1.959	− 6.2
Borneol	ILE559	Hydrogen bond	2.177	− 6.1
	ALA366	Hydrophobic	4.791	
Terpinen-4-ol	ALA366	Hydrophobic	5.141	− 5.7
	ILE559	Hydrophobic	5.35	
Thymol	LEU365	Hydrogen bond	2.603	− 5.9
	ILE416	Hydrogen bond	2.495	
	ALA556	Hydrophobic	3.774	
	ARG415	Hydrophobic	3.968	
	ARG415	Hydrophobic	5.144	
	ALA556	Hydrophobic	5.218	
Caryophyllene	ALA366	Hydrophobic	4.179	− 7.5
Himachalene	ALA366	Hydrophobic	4.797	− 7.1
Caryophyllene oxide	ALA366	Hydrophobic	4.941	− 7.9
*Trans*-cadinol	ILE559	Hydrogen bond	2.013	− 7.0
	CYS368	Hydrophobic	5.128	
	ALA466	Hydrophobic	4.839	
	VAL467	Hydrophobic	5.439	
	ALA607	Hydrophobic	4.947	
	CYS368	Hydrophobic	4.679	
	VAL369	Hydrophobic	4.952	
	VAL420	Hydrophobic	4.333

**Table 10:** Active site interacting residues, distance (Å) and binding affinity (Kcal/mol) of phytochemical constituents of *O. grossii* with 6QME.

Interaction of EOs from *O. grossii* with 6QME	Residues	Types of interaction	Bond distance (Å)	Binding affinity (Kcal/mole)
*p*-cymene	ALA366	Hydrophobic	3.586	− 5.2
ɤ-terpinene	TYR572	Hydrophobic	3.625	− 5.2
	TYR334	Hydrophobic	4.655	
	TYR334	Hydrophobic	4.338	
	ALA556	Hydrophobic	5.320	
*Cis*-sabinene	ALA366	Hydrophobic	4.748	− 5.1
Eucalyptol	ALA366	Hydrophobic	5.327	− 5.8
α-campholenal	GLY464	Hydrogen bond	2.564	− 5.5
	ALA366	Hydrophobic	4.372	
Isoborneol	ILE416	Hydrogen bond	2.753	− 6.1
	VAL463	Hydrogen bond	2.592	
	GLY417	Hydrogen bond	3.561	
	ALA366	Hydrophobic	5.239	
Borneol	ILE559	Hydrogen Bond	2.177	− 6.1
	ALA366	Hydrophobic	4.791	
isopulegone	ALA556	Hydrophobic	3.987	− 5.7
	ARG415	Hydrophobic	3.951	
Myrtenyl acetate	ALA366	Hydrophobic	4.030	− 6.6
Carvacrol	VAL418	Hydrogen bond	3.170	− 5.9
	VAL465	Hydrogen bond	2.389	
	ALA366	Hydrophobic	3.759	
Caryophyllene	ALA366	Hydrophobic	4.179	− 7.5
ɤ- Maaliene	ALA366	Hydrophobic	4.556	− 7.4
	ALA366	Hydrophobic	4.727	
Caryophyllene oxide	ALA366	Hydrophobic	4.9411	− 7.9
Valencene	ALA366	Hydrophobic	3.831	− 7.2
co-crystallized ligand J6Q	SER508	Hydrogen bond	2.560	− 8.3
	SER602	Hydrogen bond	2.152	
	ALA556	Hydrophobic	3.779	
	TYR525	Hydrophobic	3.769	
	TYR525	Hydrophobic	4.388	
	TYR572	Hydrophobic	5.408	
	ALA556	Hydrophobic	4.137

**Figure 10:**
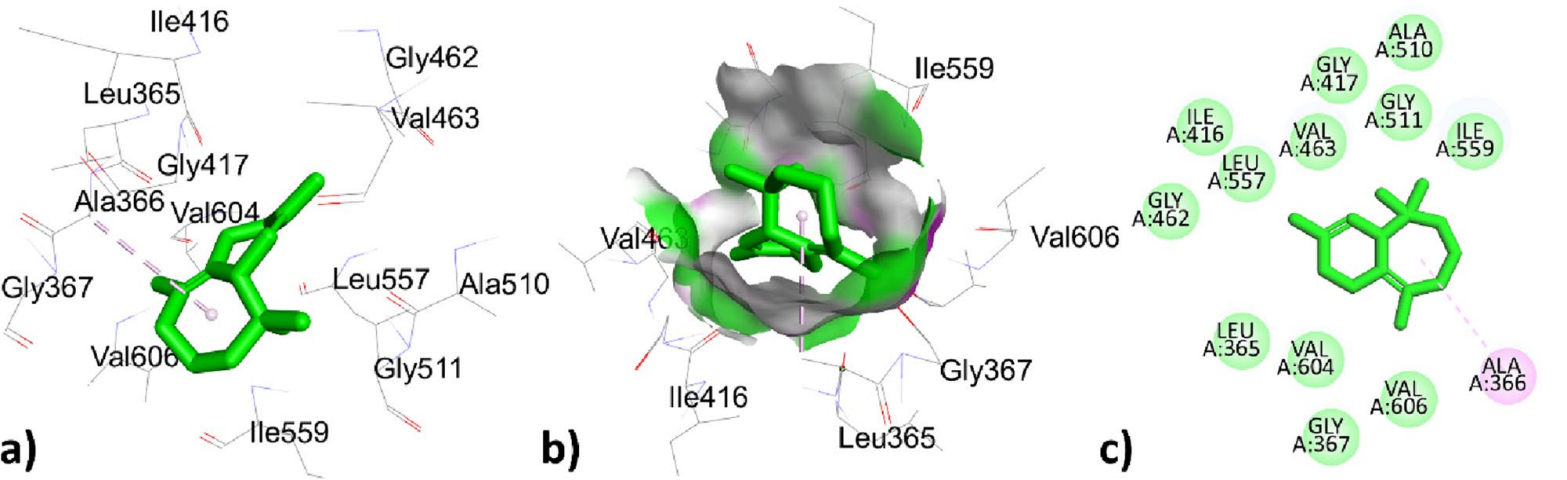
3D, hydrogen surface and 2D interaction of Himachalene with 6QME.

The 3rd highest compound from *T. pallidus* was Himachalene with binding affinity − 7.1 (Kcal/mole) and showed hydrophobic interaction to ALA366 with bond distance 4.797 (Å) represented in Fig. 10 and Table 9.

The sabinene hydrate interacted hydrophobically with amino acids ALA366, and VAL465 with distances 3.915, and 4.314 (Å); linalool oxide was hydrophically bounded to residue ILE416 with a distance of 1.959 (Å); Terpinen-4-ol also interacted through the hydrophobic bond to ALA366, ILE559 with bond distance 5.141, 5.35 (Å); thymol was interacted by a hydrogen bond to LEU365, ILE416 with distance 2.603, 2.495 while thymol interacted by hydrophobic interaction to residues ALA556, ARG415, ARG415, ALA556 with distance 3.774, 3.968, 5.144, 5.218 (Å); *Trans*-cadinol was interacted by hydrophobic bond CYS368, ALA466, VAL467, ALA607, CYS368, VAL369, VAL420 with bond distance 5.128, 4.839, 5.439, 4.947, 4.679, 4.952, 4.333 (Å) shown in Table 9.

Table 10 showed the interaction between the phytochemical constituents *p*-cymene, ɤ-terpinene, *cis*-sabinene, eucalyptol, α-campholenal, isoborneol, borneol, isopulegone, myrtenyl acetate, carvacrol, caryophyllene, ɤ-maaliene, valencene, caryophyllene oxide of essential oils obtained from *O. grossii* and co-crystallized ligand J6Q to antioxidant protein 6QME with docking score − 5.2, − 5.2, − 5.1, − 5.8, – 5.5, − 6.1, − 6.1, − 5.7, − 6.6, 5.9, − 7.5, − 7.4, − 7.9, − 7.2, − 8.3 respectively tabulated in Table 10. The *cis*-sabinene and eucalyptol, myrtenyl acetate, and valencene were bounded by hydrophobic bonds to ALA366 with bond distances 3.586, 5.327, 4.030, 3.831 (Å) respectively shown in Table 10.

The α-campholenal was bounded through hydrogen bond interaction to GLY464 and bounded hydrophobically to ALA366 with distances 2.564, and 4.372 (Å) respectively shown in Table 10. The Isoborneol represented two types of interactions, one was by hydrogen bond to residues ILE416, VAL463, GLY417 with bond distance 2.753, 2.592, 3.561 (Å) and interacted hydrophobically to residues ALA366 with bond distance 5.239 (Å) tabulated in Table 10.

The isopulegone showed the hydrophobic interaction to residues ALA556, and ARG415 with bond distances 3.987, 3.951 (Å); carvacrol was bounded through hydrogen bonds to residues VAL418, and VAL465 with bond distances 3.170, 2.389 (Å) and showed hydrophobic interaction to residues ALA366 with bond distance 3.759 (Å) shown in Table 10.

**Figure 11:**
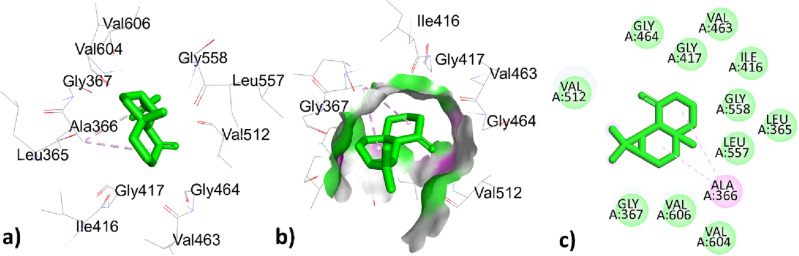
3D, hydrogen surface and 2D interaction of ɤ-Maaliene with 6QME.

The first two highest top-ranked constituents of *O. grossii* with 6QME were already discussed above and represented in Figs. 6 and 8; while the third highest top-ranked compound ɤ-Maaliene with docking score − 7.4 (Kcal/mole) showed hydrophobic interaction to residues ALA366 with bond distance 4.556, 4.727 (Å) represented in Fig. 11 and Table 10.

**Figure 12:**
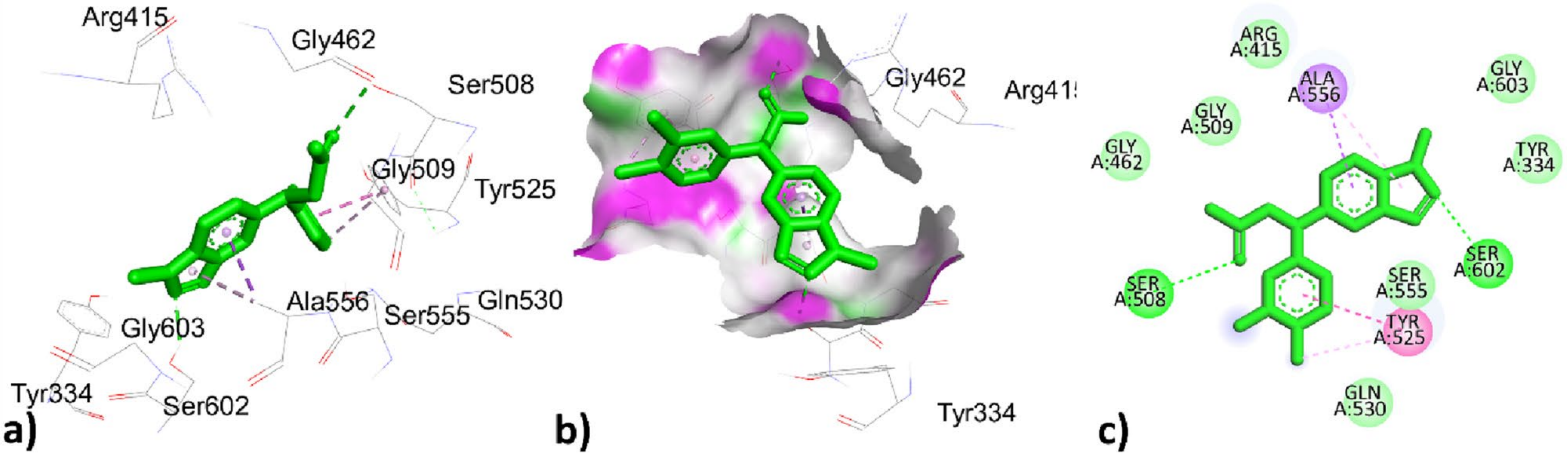
3D, hydrogen surface and 2D interaction of co-crystallized ligand J6Q with 6QME.

The co-crystallized ligand J6Q showed two types of interactions, one was hydrogen bond to residues SER508, and SER602 with bond lengths 2.560, 2.152 (Å) while the other was hydrophobic interactions to residues ALA556, TYR525, and TYR525, TYR572, ALA556 with bond distance 3.779, 3.769, 4.388, 5.408, 4.137 with docking score − 8.3 (Kcal/mole) which was closely related to the highest 3 top ranked constituents of *O. grossii* and *T. pallidus* plants Fig. 12 and Table 10.

## Discussion

The yields of both plants were higher compared to previous studies on similar species, which reported yields of 2% and 0.26% for *O. grossii* and *T. pallidus*, respectively^9,12^. The determination of the chemical constituents and flavonoid concentration of EOs holds considerable significance in the taxonomic classification and differentiation of various thyme species^53^. Notably, in *T. pallidus*, thymol emerged as the predominant compound, constituting a substantial proportion of 86% (Table 1). However, an alternative study reported a distinctive composition, with borneol representing the major constituent at 41.67%^54^. Furthermore, another study on the EOs reported the presence of 15 compounds, with γ-Terpinene emerging as the principal component, comprising 29.6% of the total composition^55^. For *O. grossii*, it is worth noting that although the primary component remained consistent, *Figueredo*^56^ observed structural variations in the chemical profile of *O. grossii* EO, suggesting inherent heterogeneity^56^. This heterogeneity in EO composition within the same species can be attributed to diverse factors, including environmental conditions, geographical location, timing of harvest, and variations in distillation techniques. Notably, climatic factors have been identified as particularly influential contributors^57^.

Antioxidant activity was evaluated through DPPH scavenging ability, Ion reducing ability and Total Antioxidant Activity. For DPPH activity, noteworthy, limited literature is available concerning *O. grossii* EO. Conversely, the EOs of *T. pallidus* exhibited a higher antioxidant activity compared to the findings reported by Laila El Bouzidi (IC_50_ = 345.11 ± 7.46 μg/mL)^55^, as well as *Thymus vulgaris* (IC_50_ = 0.259 ± 0.476 μg/mL) as reported in^58^.

As regards the ferric-reducing power capacity assay was employed to investigate the redox-modulating potential of EOs, as well as their ability to neutralize reactive species^59^. The underlying principle of this assay involves the reduction of hydroxyl radicals generated by the interaction of Fe_2_ + and H_2_O_2_, facilitated by the antioxidants present in the EOs, which subsequently chelate the resulting Fe_2_ + hydroxyl radicals^60^. Interestingly, the values of our test surpassed those reported for the EOs of other *Origanum* and *Thymus* species, including *Origanum Vulgaris*, *T. satureioides*, *T. maroccanus*, and *T. broussonetii* EOs, as documented by^55,61^. However, it is pertinent to note that the antioxidant activity of the tested EOs was notably lower than that of the pure reference antioxidant Quercetin (0.03 g/mL)^62–64^.

The total antioxidant capacity (TAC) of the investigated EOs, reference antioxidants, and BHT was assessed using the phosphomolybdenum method, as detailed by Pilar Prieto et al.^25^. Noteworthy, the methodology involves the conversion of Mo (VI) to Mo (V) in the presence of an antioxidant compound. The determination of TAC serves as a crucial parameter to evaluate the capacity of a substance to counteract unwanted oxidation processes. It holds biological significance by providing insights into the antioxidative strength exhibited by the studied substance. The antioxidant potential of phenolic compounds is attributed to the presence of hydroxyl groups, which confer their scavenging ability towards free radicals. Notably, a higher content of hydroxyl groups within these compounds corresponds to an enhanced capability to neutralize reactive species^21,65^.

The antioxidant property of phenolic compounds is closely linked to their structural features, which often include functional groups capable of binding and neutralizing free radicals^66^. Comparing the TPC and TFC results of *T. pallidus* in our study to those reported in the literature for *Thymus* species like *T. vulgaris* (19.2 ± 0.3 mg GAE/g)^66^, we observed that *T. pallidus* exhibited higher values. Moreover, *T. daenensis* subsp. and *T. kotschyanus* demonstrated TPC values of 295.93 ± 34.07 mg GAE/g and 337.00 ± 8.31 mg GAE/g, respectively^67^. Regarding TFC, *T. capitatus* displayed a flavonoid concentration of 10.62 ± 0.24 mg QE/g^68^.

Given that phenolic compounds often contain molecules that effectively bind and counteract free radicals, the antioxidative characteristic is inherently linked to its phenolic structure^66^. Consequently, to evaluate the antioxidant activity of *O. grossii* and *T. pallidus* essential oils, we examined the total flavonoid content (TFC) and total phenolic content (TPC) of each test sample. The results of this evaluation are presented in Table 4. TFC results were standardized against quercetin as a reference, and the outcomes were expressed in terms of mg Q E (milligrams of quercetin equivalents) per gram of essential oil, as shown in Table 4. Similarly, the findings of the comparison of TPC against the standard gallic acid were reported as mg GAE (milligrams of gallic acid equivalents) per gram of the sample. Notably, the essential oil from *O. grossii* exhibited the highest total flavonoid and phenolic content, measuring 0.207 ± 0.007 mg Q E/g EO and 0.136 ± 0.13 mg GAE/g EO, respectively^69–71^.

The analysis of Pearson’s Correlation revealed significant correlations between these variables. The total flavonoid content (TFC) exhibited strong positive correlations with the total antioxidant capacity (TAC) assay (r = 0.977), indicating that the flavonoids present in the samples contribute significantly to their antioxidative effects. Conversely, TFC showed negative linear correlations with the DPPH assay and the FRAP assay (r = − 0.997 and − 0.981, respectively), suggesting an inverse relationship between TFC and the scavenging of DPPH radicals and the reducing power of the samples. Furthermore, the DPPH assay demonstrated the highest correlation with the total phenolic content (TPC) among the antioxidant tests (r = − 0.852), indicating that polyphenols play a crucial role in the observed antioxidant activity measured by the DPPH assay. It is important to note that different phenolic compounds may exhibit varying responses to different antioxidant mechanisms, leading to variations in correlation strengths depending on the specific antioxidant assay employed. These findings highlight the significance of flavonoids and polyphenols in the antioxidant properties of the *O. grossii* and *T. pallidus* samples. The correlations observed between the total phenolic and flavonoid contents and the different antioxidant assays provide valuable insights into the specific mechanisms and compounds responsible for the observed antioxidant activity.

The antibacterial activity of *O. grossii* and *T. pallidus* EOs was evaluated against four bacterial strains: *Salmonella sp*, *Streptococcus sp*, *S. aureus*, and *E. coli*, which are known to be responsible for foodborne infections. These bacterial strains were obtained from the Center of the Hospital University of Fez, Morocco, and stored in the regional laboratory of epidemiological diagnosis and hygiene in Fez. The potent antibacterial activity of *O. grossii* and *T. pallidus* EOs can be attributed to their rich compositions of carvacrol and thymol, respectively. Previous studies investigating the antibacterial properties of *Lippia sidoides* EO and its main components, thymol, and carvacrol, have demonstrated their strong inhibitory effects against bacteria and fungi^72,73^. These findings highlight the potential of *O. grossii* and *T. pallidus* EOs as natural antibacterial agents due to their chemical compositions and strong inhibitory effects against both Gram-positive and Gram-negative bacteria^74,75^. Since Eos’ mechanism of action and its constituents (thymol and carvacrol) are hydrophobic, they may interact with the lipid bilayer of bacterial cytoplasmic membranes, causing a loss of integrity, increasing the fluidity and permeability of the membrane, and allowing cellular components like ions, ATP, and nucleic acids to leak out^75^.

## Conclusions

Our study focused on the antioxidant capacity based on DPPH radical scavenging activity, CAT, FRAP, TPC, and TFC of that *O. grossi* and *T. pallidus*, and the antibacterial effect against bacteria species causing food poisoning. The results of this study suggest that the chemicals in *O. grossii* and *T. pallidus* plant EOs have antibacterial and antioxidant properties. These findings could lead to the development of new natural products with antibacterial and antioxidant activity. The chemicals in *O. grossii* that were found to have antibacterial activity were *p*-cymene, eucalyptol, and carvacrol. These chemicals are known to work by disrupting the cell membranes of bacteria, which can lead to cell death. The chemicals in *T. pallidus* that were found to have antibacterial activity were *p*-cymene, α-terpinene, and thymol. These chemicals are also known to work by disrupting the cell membranes of bacteria. The chemicals in both plants that were found to have antioxidant activity were *p*-cymene, eucalyptus, carvacrol, α-terpinene, and thymol. These chemicals are known to work by scavenging free radicals, which can damage cells. The findings of this study suggest that the chemicals in the studied EOs could be used to develop new natural products with antibacterial and antioxidant activity. These products could be used to treat a variety of conditions, including infections and inflammation.

## Data Availability

All data generated or analyzed during this study are included in this published article.
